# Prioritization: Intracranial Hemorrhage, Testicular Torsion, and Tricyclic Antidepressant Toxicity Presenting to a Community Emergency Department

**DOI:** 10.21980/J8.52346

**Published:** 2025-12-31

**Authors:** Brian Milman, Marshall Howell, Joshua Ginsburg, Samuel Parnell

**Affiliations:** * University of Texas Southwestern Medical Center, Department of Emergency Medicine, Dallas, TX

## Abstract

**Audience:**

This case is designed for emergency medicine residents preparing for the American Board of Emergency Medicine Certifying Exam (ABEM). While we tested the case with third year emergency medicine residents, it could also be used with first- and second-year residents to develop complex decision-making and prioritization skills in a simulated environment.

**Introduction:**

Emergency medicine requires physicians to rapidly prioritize care, stabilize critically ill patients, adapt to changing clinical circumstances, and delegate tasks and resources. Traditional oral board cases emphasize single-patient encounters rather than multitasking or task-switching. This prioritization case better aligns with the clinical workflow of a shift in the emergency department, including triage, teamwork, and flexibility. This case forces learners to make timely decisions with incomplete information, giving examiners insight into how the examinee performs in the clinical environment.

**Educational Objectives:**

By the end of this case learners should: 1) Become familiar with the format of a prioritization case (a component of the ABEM Certifying Exam), 2) demonstrate their ability to prioritize multiple patients and provide stabilizing care, 3) consider changes in status/patient acuity/new cases as presented, 4) understand how to utilize team resources appropriately.

**Educational Methods:**

A group of five emergency medicine faculty with experience in simulation and oral board case design created a 15-minute practice prioritization case. This case is based on information provided by ABEM on the prioritization case format from the ABEM Certifying Exam. Learners are presented with evolving patient scenarios via tracking boards and prompted to prioritize, stabilize, task switch, and delegate as they manage multiple patients. The case is intended to be administered with two examiners and one examinee at a time. We used a group debrief structure, but this case can also be debriefed with each individual learner.

**Research Methods:**

This case was tested on 18 third-year emergency medicine residents. Following the case, each resident completed an anonymous two-item evaluation. The first item, “This case increased my understanding of the certifying exam format,” was scored on a 5-point Likert scale from “strongly disagree” to “strongly agree.” The second item, “How would you rate the overall quality of this case?” was scored on a 5-point Likert scale from “poor” to “excellent.”

**Results:**

Sixteen of eighteen (89%) examinees completed the post-case evaluation. All respondents (100%) “agreed” or “strongly agreed” that the case improved their understanding of the ABEM Certifying Exam format. Overall case quality was rated 4.88/5, and all learners rated the case “very good” or “excellent.”

**Discussion:**

This case was effective in simulating the competing demands of the clinical environment while also preparing learners for a new exam format. During the group debrief session, learners appreciated the pace, needing to stabilize multiple patients, and reacting to new information as it was presented. This case significantly improved residents’ understanding of the prioritization case type that will be tested on the ABEM Certifying Exam. It also provides a controlled environment for program faculty to observe how residents perform managing multiple sick patients simultaneously.

**Topics:**

Prioritization, resource utilization, triage and stabilization, task-switching, cognitive load.

## USER GUIDE

**Table:** 

List of Resources:	
Abstract	370
User Guide	372
For Examiner Only	374
Certifying Exam Assessment	380
Stimulus	387
Debriefing and Evaluation Pearls	396

**Learner Audience:**

This case is appropriate for interns and junior and senior residents.

**Time Required for Implementation:**

Case: Prioritization cases are 15 min by the American Board of Emergency Medicine (ABEM) standard

Debriefing: 10-minute group debrief.

**Recommended number of learners per instructor:**

One learner paired with two examiners

**Topics:**

Prioritization, resource utilization, triage and stabilization, task-switching, cognitive load.

**Objectives:**

By the end of this structured interview case, learners will be able to:

1. Demonstrate familiarity with the certifying exam prioritization case format and case flow.
2. Prioritize multiple undifferentiated patients in a resource-limited emergency department setting using limited triage-level information.
3. Adapt clinical priorities dynamically as new diagnostic information is presented and new high-acuity patients arrive.
4. Demonstrate effective task delegation to ensure that emergent interventions are performed in a timely manner.

### Linked objectives, methods and results

In the ABEM Certifying Exam, this clinical case is a structured discussion with two examiners. This practice case may be done with one or two examiners.

This case will assess the ability to prioritize multiple patients in the emergency department or prehospital setting and provide stabilizing care.

The successful candidate will:

- Determine acuity of patient(s)
- Provide appropriate and immediate stabilizing care
- Respond to changes in patient acuity and triage new cases as presented
- Use team resources appropriately

1. Patient Prioritization and Initial Assessment:
  - Analyze a patient tracking board and determine the most critical patients based on limited initial information.
  - Identify key history, physical exam findings, and immediate diagnostic tests needed for rapid decision-making.
2. Stabilization and Initial Management:
  - Implement appropriate immediate interventions for critically ill patients, such as hypertension management, anticoagulation reversal, analgesia, and procedural intervention.
  - Recognize and manage time-sensitive conditions, including central nervous system (CNS) pathology, testicular torsion, and tricyclic antidepressant (TCA) toxicity.
3. Task Delegation & Team Management:
  - Identify tasks that can be delegated to the healthcare team to optimize workflow and efficiency.
  - Communicate clear and concise instructions to support staff during patient management.
4. Dynamic Adaptation and Task Switching:
  - Adjust clinical priorities based on additional diagnostic data and evolving patient conditions.
  - Reprioritize care as new patients arrive, balancing initial assessments with ongoing interventions.
5. Procedural Decision-Making and Execution:
  - Determine the appropriate use of analgesia and procedural interventions for acutely ill patients.
  - Perform or direct critical procedures such as manual detorsion and vascular access placement.
6. Case Completion and Reflection (Debrief):
  - Review and reflect on decision-making strategies at the conclusion of the case.
  - Identify areas for improvement in prioritization, delegation, and procedural execution.

Traditionally, most of the cases tested on the ABEM Oral Exam were single patient encounters. The single patient encounter case does not test proficiency in ACGME Milestone PC7: Multitasking (task-switching). The new certifying exam format incorporates a prioritization case that will require examinees to determine the acuity of multiple patients, provide immediate stabilizing care, respond to changes in acuity, triage new patients, and use team resources effectively. We utilized a naturalistic decision-making (NDM) framework to develop this prioritization case. The NDM framework studies how people make decisions and perform in complex environments. The ED is a dynamic, high-stakes environment that requires physicians to prioritize patients, care for multiple patients simultaneously, and involve team members to accomplish additional tasks. These skills are not easily assessed in a testing environment. The NDM framework allowed us to best simulate the ED clinical environment by forcing learners to make decisions with incomplete information and to respond to realistic disruptions.

### Recommended pre-reading for instructor

- The American Board of Emergency Medicine. Certifying Exam Content. Prioritization Sample Case Video. Accessed November 8, 2025. https://www.abem.org/get-certified/certifying-exam/certifying-exam-content/
- The American Board of Emergency Medicine. Certifying Exam Content. Prioritization Debrief Video. Accessed November 8, 2025. https://www.abem.org/get-certified/certifying-exam/certifying-exam-content/

### Results and tips for successful implementation

This case was developed by a team experienced in resident education, simulation case development, and oral exam case development. Prior to administering the case with residents, the case was piloted with one third-year resident from another institution, one fellow from our institution, and one faculty member. Some vital signs were adjusted following piloting to make the case presentations clearer, and a minor modification was made to the script. Otherwise, no changes were made. We implemented this case during a Mock Certifying Exam Day for third-year EM residents, which allowed for an experience that closely resembled the environment of the actual exam. However, this case could easily be utilized in isolation rather than as part of a structured day of examinations. The case was then administered to 18 third-year residents. Two faculty members administered the case to one resident at a time. Of the 18 residents, 16 (88.89%) completed a post-case evaluation. When asked if this case increased understanding of the certifying exam format, 16 (100%) agreed or strongly agreed. When asked about the overall quality of the case, 16 (100%) responded “very good” or “excellent.” This case received a score of 4.88/5 for overall quality.

In a group debrief, after all learners had completed the case, there was no feedback from learners that necessitated additional modifications to the case. Overall, the residents felt that the scenarios were realistic and thought that this case type was representative of flow in the clinical environment. Recommendations from faculty who administered the case included building additional structure into the feedback form to capture some of the history, diagnostics, and actions that the learner performs during the case since there are multiple scoring criteria for the examiners to manage simultaneously.

## Figures and Tables

**Table:** PRIORITIZATION (PROMPT 1, 2, 6, 8)

Patient	Age	Chief Complaint	Vital Signs	Priority	Checklist
A	45 y/o man	Chest pain	BP: 144/86 P: 86 R: 16 Temp: 37.0° C (98.6° F) Sat: 98% on room air	5	□ Medical history □ Cardiac exam □ ECG
B	77 y/o woman	Headache	BP: 182/90 P: 61 R: 18 Temp: 37.0° C (98.6° F) Sat: 96% on room air	2 (but becomes most critical patient after CT is performed).	□ Anticoagulation use □ Neuro exam
C	14 y/o boy	Testicular pain	BP: 116/74 P: 114 R: 20 Temp: 37.0° C (98.6° F) Sat: 98% on room air	1	□ Time of onset □ Testicular exam
D	19 y/o man	Shortness of breath	BP: 126/80 P: 71 R: 24 Temp: 37.0° C (98.6° F) Sat: 97% on room air	4	□ Medical history □ Work of breathing □ Pulmonary exam
E	23 y/o woman	Vaginal bleeding	BP: 127/84 P: 116 R: 16 Temp: 37.0° C (98.6° F) Sat: 98% on room air	3	□ Quantify bleeding □ Urine pregnancy □ Abdominal exam □ History of coagulopathy
F	58 y/o woman	Hyperglycemia	BP: 151/81 P: 75 R: 16 Temp: 37.0° C (98.6° F) Sat: 99% on room air	6	□ POC Glucose □ Medical history
G	51 y/o woman	Ingestion	BP: 101/65 P: 126 R: 14 Temp: 38.0° C (100.4° F) Sat: 92% on room air	G should be seen before H	
H	33 y/o man	Knee pain	BP: 124/80 P: 75 R: 16 Temp: 37.0° C (98.6° F) Sat: 99% on room air		

**Table:** STABILIZATION (PROMPTS 2, 4, 6, 7, 8)

Patient	Age	Chief Complaint	Vital Signs	Actions/Procedures
B (Prompt 4)	77 y/o woman	Headache	BP: 182/90 P: 61 R: 18 Temp: 37.0° C (98.6° F) Sat: 96% on room air	□ IV antihypertensive □ Reverse coagulopathy □ Consult neurosurgery
C (Prompt 7)	14 y/o boy	Testicular pain	BP: 116/74 P: 114 R: 20 Temp: 37.0° C (98.6° F) Sat: 98% on room air	□ Analgesia □ Manual detorsion □ Transfer to a receiving hospital with urology
G (Prompt 8)	51 y/o woman	Ingestion	BP: 101/65 P: 126 R: 14 Temp: 38.0° C (100.4° F) Sat: 92% on room air	□ Sodium bicarb □ Consult poison center or toxicology

**Table:** DELEGATON (PROMPT 5)

The successful candidate will delegate 4 or more of the tasks below to optimize care. The candidate should receive credit for the number of tasks that they delegate if they delegate fewer than 4 tasks. If they delegate more than 4 tasks, no additional credit is given. IV insertion for patients A, B, C, E, G Draw and hold blood for A, B, E, G Initiating transfer process for patient C Arranging ambulance or helicopter EMS for patient C Calling poison center for patient G Paging neurosurgery for patient B Repeat respiratory exam for patient D Repeat vital signs for patient E Repeat pain assessment for patients A, E Calling radiology department to expedite patient E’s ultrasound

**Table:** TASK SWITCHING (PROMPTS 3, 8)

Patient	Age	Chief Complaint	Vital Signs	Actions/Procedures
B (Prompt 6)	77 y/o woman	Headache	BP: 182/90 P: 61 R: 18 Temp: 37.0° C (98.6° F) Sat: 96% on room air	□ Following Prompt X, Patient B becomes the most critical patient and should be immediately addressed
G (Prompt 9)	51 y/o woman	Ingestion	BP: 101/65 P: 126 R: 14 Temp: 38.0° C (100.4° F) Sat: 92% on room air	□ Following arrival of G and H, patient G should be immediately assessed

**Table:** 

□ Reviewing Patient B’s head CT immediately following the phone call from the radiology tech	1-point
□ Evaluating Patient B after diagnosing intracranial hemorrhage	2-point
□ Evaluating Patient G as soon as she arrives	1-point

**Table:** 

Domain	Score	Strengths	Growth Opportunities
Patient Prioritization Questions	___ / 12 * 0.25		
Stabilization & Initial Management	___ / 9 * 0.25		
Task Delegation & Team Management	___ / 4 * 0.125		
Task Switching & Adaptation	___ / 4 * 0.125		
Prioritization Order	((36-____/36) * 0.25		
**Total %**	*_________*		

**Table:** 

**Candidate Task Sheet**	
**#1**	**Tracking Board #1**
**#2**	**Tracking Board #2**
**#3**	**ECG – Patient A**
**#4**	**CT Brain – Patient B**
**#5**	**Tracking Board #3**
**#6**	**ECG – Patient G**

**Table:** Prioritization Candidate Task Sheet

**CASE PARAMETERS**
This is a 15-minute case. You will interact with two examiners. This is an interview style case without role playing. You will evaluate and treat multiple patients while ensuring those who require immediate care receive it as quickly as possible. You will have two nurses to complete orders and assist you in basic assessment of patients. You may face the arrival of additional patients, deterioration of existing patients, and workflow interruptions during the case.
**PATIENT INFORMATION**
Relevant information will be provided on your tracking board. Given multiple patients, you will be asked to identify individual features that would help you determine each patient’s acuity.
**TASK STATEMENT**
Your tasks are as follows: Given multiple patients, you will be asked to identify individual features that would help you determine each patient’s acuity. Identify the patients that most urgently require medical attention and provide stabilizing care. Effectively manage available clinical resources. Provide care in the setting of changing clinical conditions including new patients, changes in acuity, critical diagnostic results, and limitations of resources.

**STIMULUS 1:** Tracking Board #1

Patient	Age	Chief Complaint	Vital Signs
A	45 y/o man	Chest pain	BP: 144/86 P: 86 R: 16 Temp: 37.0° C (98.6° F) Sat: 98% on room air
B	77 y/o woman	Headache	BP: 182/90 P: 61 R: 18 Temp: 37.0° C (98.6° F) Sat: 96% on room air
C	14 y/o boy	Testicular pain	BP: 116/74 P: 114 R: 20 Temp: 37.0° C (98.6° F) Sat: 98% on room air
D	19 y/o man	Shortness of breath	BP: 126/80 P: 71 R: 24 Temp: 37.0° C (98.6° F) Sat: 97% on room air
E	23 y/o woman	Vaginal bleeding	BP: 127/84 P: 116 R: 16 Temp: 37.0° C (98.6° F) Sat: 98% on room air
F	58 y/o woman	Hyperglycemia	BP: 151/81 P: 75 R: 16 Temp: 37.0° C (98.6° F) Sat: 99% on room air

**STIMULUS 2:** Tracking Board #2

Patient	Age	Chief Complaint	Vital Signs	Condition Report
A	45 y/o man	Chest pain	BP: 144/86 P: 86 R: 16 Temp: 37.0° C (98.6° F) Sat: 98% on room air	No cardiac risk factors. Normal exam. ECG stimulus available.
B	77 y/o woman	Headache	BP: 182/90 P: 61 R: 18 Temp: 37.0° C (98.6° F) Sat: 96% on room air	Medications include apixaban. Follows commands. Protecting airway. CT head ordered.
C	14 y/o boy	Testicular pain	BP: 116/74 P: 114 R: 20 Temp: 37.0° C (98.6° F) Sat: 98% on room air	Uncomfortable appearing. Began 2 hours ago. Swollen right testicle. Absent right cremasteric reflex. This facility has no pediatric specialists.
D	19 y/o man	Shortness of breath	BP: 126/80 P: 71 R: 24 Temp: 37.0° C (98.6° F) Sat: 97% on room air	History of asthma. Out of home inhaler. Appears comfortable. End expiratory wheezes.
E	23 y/o woman	Vaginal bleeding	BP: 127/84 P: 116 R: 16 Temp: 37.0° C (98.6° F) Sat: 98% on room air	Bleeding began yesterday. 4 pads in the past 24 hours. HCG positive. Ultrasound ordered.
F	58 y/o woman	Hyperglycemia	BP: 151/81 P: 75 R: 16 Temp: 37.0° C (98.6° F) Sat: 99% on room air	Asymptomatic. Blood glucose – 117 mg/dL.

**STIMULUS 3:**
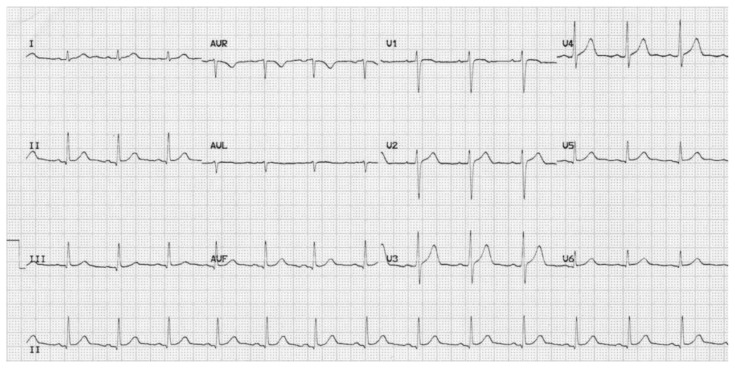
ECG – Patient A^8^

**STIMULUS 4:**
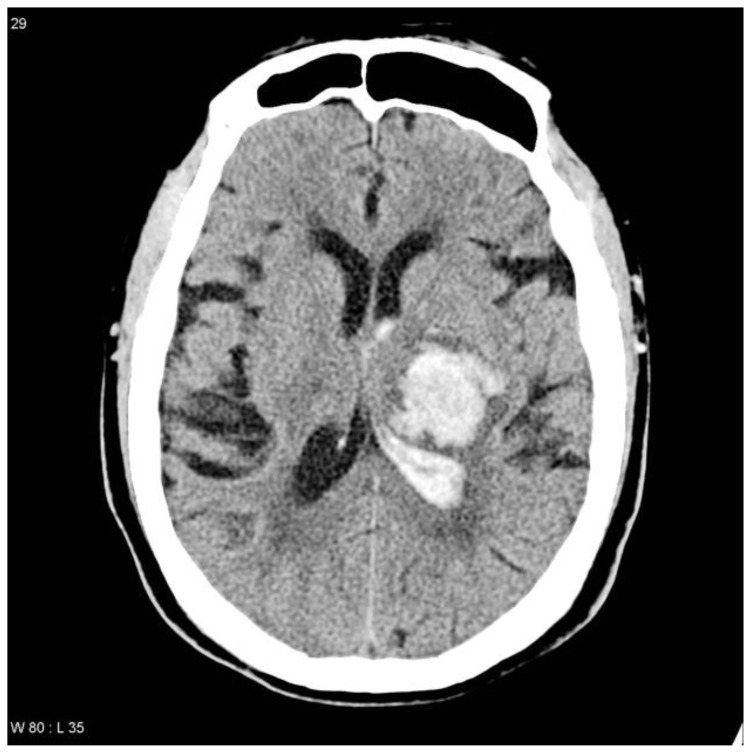
CT Brain – Patient B^9^

**STIMULUS 5:** Tracking Board #3

Patient	Age	Chief Complaint	Vital Signs	Condition Report
A	45 y/o man	Chest pain	BP: 144/86 P: 86 R: 16 Temp: 37.0° C (98.6° F) Sat: 98% on room air	No cardiac risk factors. Normal exam. ECG stimulus available.
B	77 y/o woman	Headache	BP: 182/90 P: 61 R: 18 Temp: 37.0° C (98.6° F) Sat: 96% on room air	Medications include apixaban. Follows commands. Protecting airway. CT head ordered.
C	14 y/o boy	Testicular pain	BP: 116/74 P: 114 R: 20 Temp: 37.0° C (98.6° F) Sat: 98% on room air	Uncomfortable appearing. Began 2 hours ago. Swollen right testicle. Absent right cremasteric reflex. This facility has no pediatric specialists.
D	19 y/o man	Shortness of breath	BP: 126/80 P: 71 R: 24 Temp: 37.0° C (98.6° F) Sat: 97% on room air	History of asthma. Out of home inhaler. Appears comfortable. End expiratory wheezes.
E	23 y/o woman	Vaginal bleeding	BP: 127/84 P: 116 R: 16 Temp: 37.0° C (98.6° F) Sat: 98% on room air	Bleeding began yesterday. 4 pads in the past 24 hours. HCG positive. Ultrasound ordered.
F	58 y/o woman	Hyperglycemia	BP: 151/81 P: 75 R: 16 Temp: 37.0° C (98.6° F) Sat: 99% on room air	Asymptomatic. Blood glucose – 117 mg/dL.
G	51 y/o woman	Ingestion	BP: 101/65 P: 126 R: 14 Temp: 38.0° C (100.4° F) Sat: 92% on room air	Found next to empty bottle of amitriptyline. Minimally responsive. ECG stimulus available.
H	33 y/o man	Knee pain	BP: 124/80 P: 75 R: 16 Temp: 37.0° C (98.6° F) Sat: 99% on room air	No signs of trauma. No signs of infection. Normal neurovascular exam.

**STIMULUS 6:**
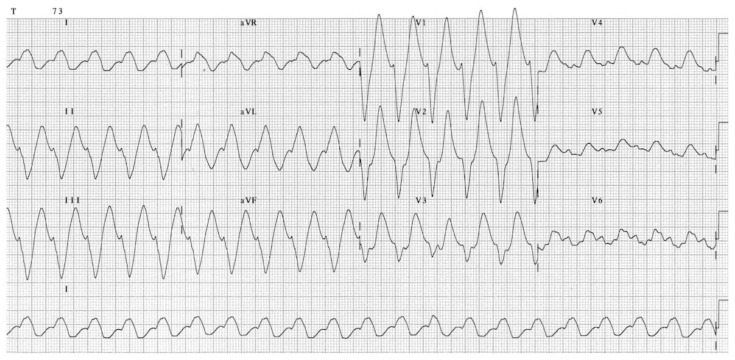
ECG – Patient G^10^

## FOR EXAMINER ONLY

### Prioritization: Intracranial Hemorrhage, Testicular Torsion, and Tricyclic Antidepressant Toxicity Presenting to a Community Emergency Department Examiner Script

For examiner: After each prompt, pause for the examinee response and record their answer on the Scoring Sheet. Scoring guidelines/answers are provided under each prompt.

Text that should be read to the examinee is highlighted in yellow.

Text that prompts release of is highlighted in blue.

Text that is not highlighted is to help the examiner.

**Case Introduction:**

“Hello doctor, come on in. Welcome, please take a seat. My name is Dr. [YOUR NAME] and I will be your examiner today. This is a prioritization case. Keep in mind that while prioritizing care, patient information and diagnostic results provided by the examiner may be very limited. I may interrupt you to move through the case. This does not reflect your performance. In the time allotted, it's possible you may not have the opportunity to completely manage every patient on your tracking board.

“Here is your task sheet for this case:”

*Show Prioritization Candidate Task Sheet*

“You will have 15 minutes to complete the case. Before we begin, do you have any questions?”

“The clock starts now.”

***Note for examiner: We strongly recommend setting a timer.

#### PRIORITIZATION

“You are a single-coverage ED physician working at a community hospital. Six patients are waiting to be seen. Here is your patient tracking board. After reviewing the tracking board, let me know when you are ready to proceed.”

*Show Tracking Board #1* **[Stimulus 1]** *and pause for Examinee response.*

Prompt 1:

“What one or two most important pieces of additional information from history, physical exam, or immediately available diagnostic testing would you like to know about each patient in order for you to prioritize care?”

***Allow candidate to list additional elements of the history, physical exam, or immediately available diagnostic testing for each patient.

- If candidate lists three or more, respond “Please list only the two most important pieces of additional information you would like to know.”
- If candidate requests consultation, respond “Please list information from the history, physical exam, or immediately available diagnostic testing only.”

Scoring Guidelines:

See Scoring Sheet for Checklist

Examinees should ask for the following information (for some patients, there are three pieces of information listed; examinees are only able to ask for two pieces of information).

A – Medical/Cardiac history, cardiac exam, ECG
B – Anticoagulation use, neurologic exam
C – Time of onset, testicular exam
D – Medical history, work of breathing, lung auscultation
E – Quantify bleeding, POC urine pregnancy, abdominal exam, history of coagulopathy
F – Medical history, medication history, POC glucose

***G and H have not yet arrived, so no stratifying questions are required.

“Here is your updated patient tracking board with additional patient information. Additional diagnostic studies are highlighted in gray and are also provided. After reviewing the tracking board, let me know when you are ready to proceed.”

*Show Tracking Board #2* **[Stimulus 2]** *and pause for examinee response.*

***If candidate asks to review ECG stimulus for patient A at any point during case, provide **[Stimulus 3]**: ECG

***If candidate asks for something that is not available (eg, ECG on a patient that does not have a stimulus), report that it is normal.

***There are no pediatricians or pediatric specialists on staff at this hospital. All other specialists are available.

#### PRIORITIZATION/STABILIZATION

Prompt 2:

“Based on what you know now, which patient do you want to treat first and why?”

Scoring Guidelines:

The learner must look at Patient A’s ECG **[Stimulus 3]** prior to determining which patient to treat first to ensure that Patient A is not having a STEMI.

Patient C (14-year-old with testicular torsion) should be treated first. There is not enough information on Tracking Board #2 to suggest that Patient B (77-year-old with headache on anticoagulation) is emergent.

#### TASK SWITCHING

“Prior to going into the room, you receive a call from the radiology tech. The tech tells you that there is something abnormal on Patient B’s CT brain. The radiology report is not yet available.”

*If candidate asks to review CT: provide* **[Stimulus 4].**

Prompt 3:

“Does this change which patient you treat first?”

Scoring Guidelines:

**[Stimulus 4]** shows that Patient B has an intracranial hemorrhage. The examinee should now stabilize Patient B prior to Patient C.

#### STABILIZATION

Prompt 4:

“What immediate stabilizing treatment would you provide?”

Scoring Guidelines:

- Treat with intravenous antihypertensive medication (nicardipine, labetalol)
- Reverse anticoagulation (four-factor prothrombin complex concentrate, activated prothrombin complex concentrate, or Andexanet alfa)
- Consult neurosurgery

#### DELEGATION

Prompt 5:

“Are there any tasks for any of your other patients that you would like to delegate to your care team to do while you are caring for this patient?”

***You can remind the examinee that they have two nurses to help them (cues them to deploy resources appropriately).

*Return to Tracking Board #2* **[Stimulus 2]** *when you ask about task delegation.*

Scoring Guidelines:

At least four tasks should be delegated to the care team. If more than four tasks are delegated, no additional credit is given.

Examples of effective team utilization include delegating the following tasks:

- IV insertion for patients A, B, C, E, G
- Draw and hold blood for A, B, E, G
- Initiating transfer process for patient C
- Arranging ambulance or helicopter EMS for patient C
- Calling poison center for patient G
- Paging neurosurgery for patient B
- Repeat respiratory exam for patient D
- Repeat vital signs for patient E
- Repeat pain assessment for patients A, E
- Calling radiology department to expedite patient E’s ultrasound

#### PRIORITIZATION/STABILIZATION

Prompt 6:

“You stabilized your first patient. Which patient would you choose to evaluate second and why?”

Scoring Guidelines:

The examinee should return to Patient B (14-year-old with testicular torsion). This patient will have to be stabilized and transferred to another facility.

#### STABILIZATION

Prompt 7:

“Thank you. What immediate actions would you take?”

Scoring Guidelines:

- IV analgesia (pediatric dosing)
- Manual detorsion of right testicle
- Speak to transfer center or accepting physician at a hospital with pediatric urology

#### TASK SWITCHING/PRIORITIZATION/STABILIZATION

“You have stabilized your second patient. Before you can evaluate the remaining patients, two additional Patients (G and H) arrive by EMS and have been added to your tracking board.”

*Show Tracking Board #3* **[Stimulus 5]**

Prompt 8:

“Considering the remaining initial patients and the arrival of two new patients, who would you assess now and what immediate stabilizing treatments would you provide?”

***If candidate asks to review ECG stimulus for patient G at any point during case, *provide* **[Stimulus 6]**

Scoring Guidelines:

- Sodium bicarbonate infusion
- Poison control or toxicology consult

“All of your patients have been assessed and stabilized. Thank you, doctor. This concludes your case.”

## CERTIFYING EXAM ASSESSMENT

*Prioritization: Intracranial Hemorrhage, Testicular Torsion, and Tricyclic Antidepressant Toxicity Presenting to a Community Emergency Department*

Learner: _________________________________________

**Scoring Sheet**

The total score is as follows from the Score Sheet. Passing Total Score = 75%

Total Score = Prioritization Questions Weighted Score (Prompt 1)
  + Prioritization Order Weighted Score (Prompt 2)
  + Stabilization and Initial Management Questions Weighted Score
  + Delegation Tasks Weighted Score
  + Task Switching Weighted Score
*Total Score = ((___/12)*0.25)+ )*
  *+(((36-total displacement)/36)*0.25)*
  + *((___/9)*0.25)*
  *+ ((___/4)*0.125)*
  *+ ((___/4)*0.125*

**Patient Prioritization Questions (25%) [Prompt 1]**

○ Patient A
  ▪ Number of essential information (history, physical exam, or immediately available diagnostic testing) correctly asked *0*–*2 ______*
○ *Patient B*
  ▪ Number of essential information (history, physical exam, or immediately available diagnostic testing) correctly asked *0*–*2 ______*
○ *Patient C*
  ▪ Number of essential information (history, physical exam, or immediately available diagnostic testing) correctly asked *0*–*2 ______*
○ *Patient D*
  ▪ Number of essential information (history, physical exam, or immediately available diagnostic testing) correctly asked *0*–*2 ______*
○ *Patient E*
  ▪ Number of essential information (history, physical exam, or immediately available diagnostic testing) correctly asked *0*–*2 ______*
○ *Patient F*
  ▪ Number of essential information (history, physical exam, or immediately available diagnostic testing) correctly asked *0*–*2 ______*
○ **Prioritization Question Raw Total** ______

**Raw Score: _____ /** ***12***

**Percentage: _____**

**Prioritization Order (25%) [Prompts 1, 2, 6, 8]**

Compare the Examinee’s order to the Ideal Order. Subtract Examinee value from the Ideal Order value and take the absolute value. Add all absolute values for the total displacement and apply this equation:

**Prioritization Order Raw Total = (36-total displacement)/36**

Percentage: _____

**Stabilization & Initial Management (25%) [Prompts 2, 4, 6, 7, 8]**

1-point for each of the following critical actions:

Patient A
  □ Review ECG at any point during the case
Patient B
  □ IV antihypertensive
  □ Reverse coagulopathy
  □ Consult neurosurgery
Patient C
  □ Analgesia
  □ Manual detorsion
  □ Transfer to a receiving hospital with urology
Patient G
  □ Sodium bicarb
  □ Consult poison center or toxicology

Raw Score: _____ */ 9*

Percentage: _____

**Delegation (12.5%)** [ **Prompt 5]**

1-point for each task (max 4):

□ IV insertion for patients A, B, C, E, G
□ Draw and hold blood for A, B, E, G
□ Initiating transfer process for patient C
□ Arranging ambulance or helicopter EMS for patient C
□ Calling poison center for patient G
□ Paging neurosurgery for patient B
□ Repeat respiratory exam for patient D
□ Repeat vital signs for patient E
□ Repeat pain assessment for patients A, E
□ Calling radiology department to expedite patient E’s ultrasound

Raw Score: *_____ / 4*

Percentage: _____

**Task Switching (12.5%) [Prompts 3 & 8]**

Raw Score: *_____ / 4*

Percentage: _____

**Blank Scoring Table Notes:**

**Summative and formative comments:**

## DEBRIEFING AND EVALUATION PEARLS

### Prioritization: Intracranial Hemorrhage, Testicular Torsion, and Tricyclic Antidepressant Toxicity Presenting to a Community Emergency Department

Recommended format from SAEM Simulation Academy – EMRES.

Debrief Framework: PEARLS (Promoting Excellence and Reflective Learning in Simulation)

Objective 6: Case Completion & Reflection (Debrief):

- Review and reflect on decision-making strategies at the conclusion of the case.
- Identify areas for improvement in prioritization, delegation, and procedural execution.

Facilitator Prompts:

- Reactions (2–3 min)
  *Goal: Allow the learner to process emotional and cognitive responses.*
  - “How did that feel overall?”
  - “What was your immediate reaction to seeing all the patients on the tracking board?”
  - “Were there any moments you felt particularly confident or overwhelmed?”
- Description (3–5 min)
  *Goal: Establish a shared understanding of what happened.*
  - “Walk me through how you approached the initial board.”
  - “What was your rationale for choosing your first patient?”
  - “How did you adapt as new patients or information came in?”
